# Supplementary material for: Evolution of the Membrane Transport Protein Domain
Source: Int J Mol Sci. 2022 Jul 22;23(15):8094. doi: 10.3390/ijms23158094 (PMC9330825; doi:10.3390/ijms23158094)
Supplement: Supplementary file 1 [file ijms-23-08094-s001.zip › Figure S3.pdf]

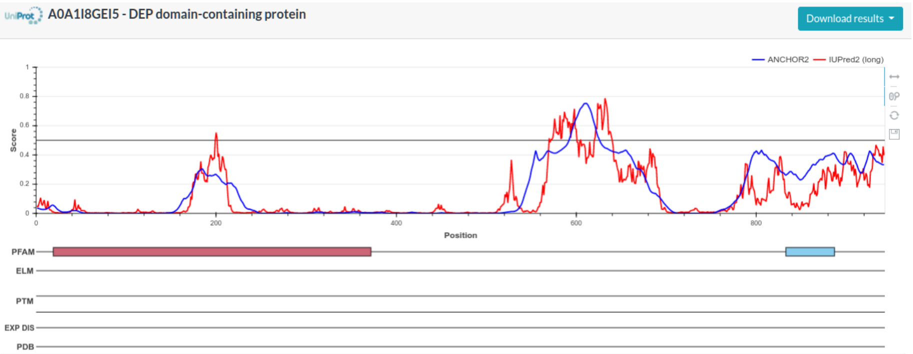

Protostomia

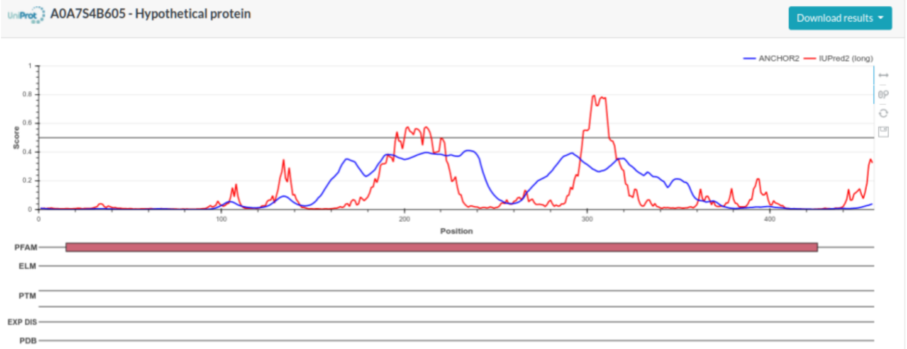

Haptista

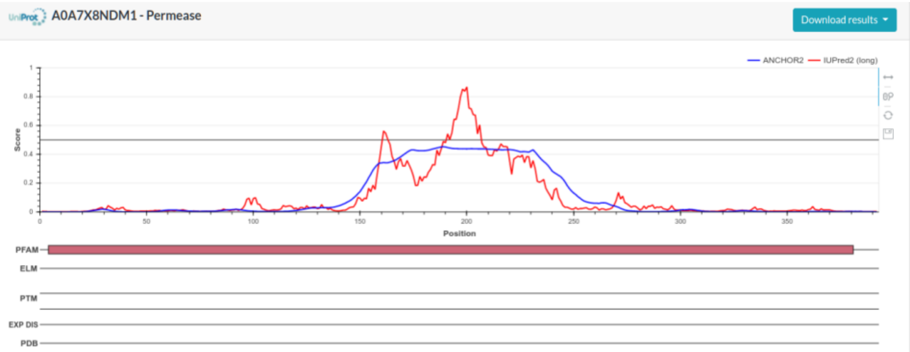

Terrabacteria,
Actinobacteria

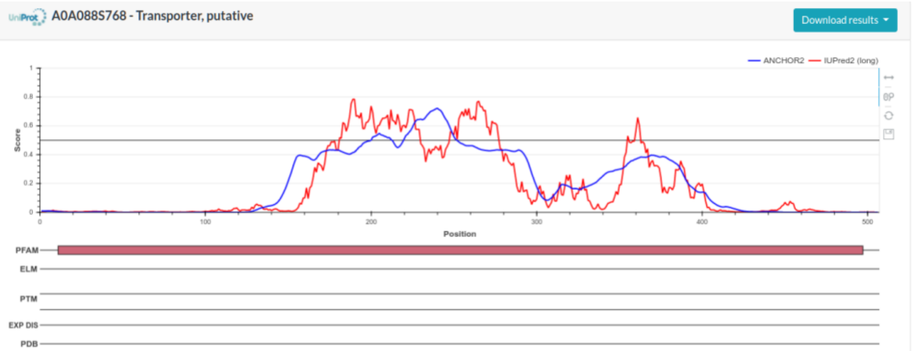

Discoba

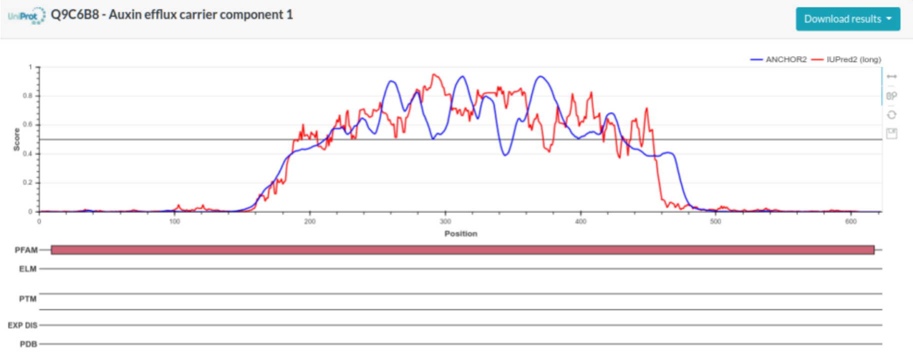

Streptophyta,
Arabidopsis thaliana
