# Supplementary material for: Evolution of the Membrane Transport Protein Domain
Source: Int J Mol Sci. 2022 Jul 22;23(15):8094. doi: 10.3390/ijms23158094 (PMC9330825; doi:10.3390/ijms23158094)
Supplement: Supplementary file 1 [file Figure S2.pdf › Figure S2.pdf]

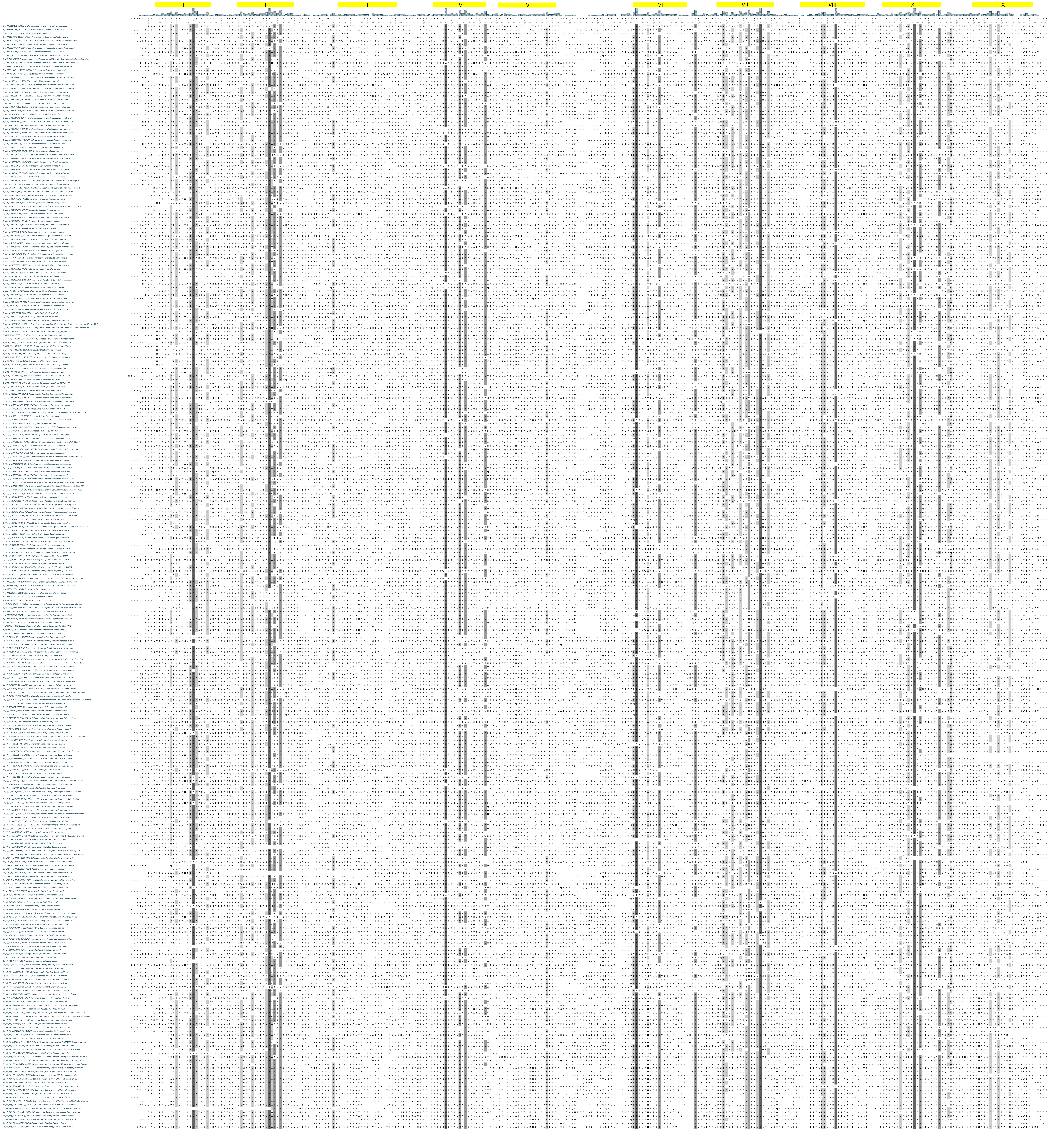
I
II
III
IV
V
VI
VII
VIII
IX
X
