# Supplementary material for: Evolution of the Membrane Transport Protein Domain
Source: Int J Mol Sci. 2022 Jul 22;23(15):8094. doi: 10.3390/ijms23158094 (PMC9330825; doi:10.3390/ijms23158094)
Supplement: Supplementary file 1 [file Figure S4.pdf › Figure S4.pdf]

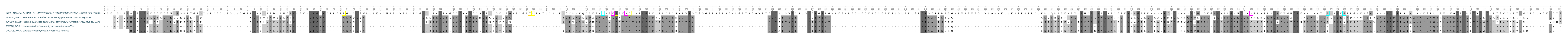
4CZ8_1|Chains A, B|NA+/H+ ANTIPORTER, PUTATIVE|PYROCOCCUS ABYSSI GE5 (272844)
F8AHV6_PYRYC Permease auxin efflux carrier family protein Pyrococcus yayanosii
I3RCU4_9EURY Putative permease auxin efflux carrier family protein Pyrococcus sp. ST04
I6UZT4_9EURY Uncharacterized protein Pyrococcus furiosus COM1
Q8U3L6_PYRFU Uncharacterized protein Pyrococcus furiosus
